# Efficient labeling for fine‐tuning chest X‐ray bone‐suppression networks for pediatric patients

**DOI:** 10.1002/mp.17516

**Published:** 2024-11-15

**Authors:** Weijie Xie, Mengkun Gan, Xiaocong Tan, Mujiao Li, Wei Yang, Wenhui Wang

**Affiliations:** ^1^ Information and Data Centre, Guangzhou First People's Hospital Guangzhou Medical University Guangzhou China; ^2^ Information and Data Centre, the Second Affiliated Hospital, School of Medicine South China University of Technology Guangzhou China; ^3^ School of Biomedical Engineering Southern Medical University Guangzhou China; ^4^ Guangdong Provincial Key Laboratory of Medical Image Processing School of Biomedical Engineering, Southern Medical University Guangzhou China

**Keywords:** bone suppression, deep learning, image processing, pediatric chest X‐ray (CXR), unsupervised learning

## Abstract

**Background:**

Pneumonia, a major infectious cause of morbidity and mortality among children worldwide, is typically diagnosed using low‐dose pediatric chest X‐ray [CXR (chest radiography)]. In pediatric CXR images, bone occlusion leads to a risk of missed diagnosis. Deep learning–based bone‐suppression networks relying on training data have enabled considerable progress to be achieved in bone suppression in adult CXR images; however, these networks have poor generalizability to pediatric CXR images because of the lack of labeled pediatric CXR images (i.e., bone images vs. soft‐tissue images). Dual‐energy subtraction imaging approaches are capable of producing labeled adult CXR images; however, their application is limited because they require specialized equipment, and they are infrequently employed in pediatric settings. Traditional image processing–based models can be used to label pediatric CXR images, but they are semiautomatic and have suboptimal performance.

**Purpose:**

We developed an efficient labeling approach for fine‐tuning pediatric CXR bone‐suppression networks capable of automatically suppressing bone structures in CXR images for pediatric patients without the need for specialized equipment and technologist training.

**Methods:**

Three steps were employed to label pediatric CXR images and fine‐tune pediatric bone‐suppression networks: distance transform–based bone‐edge detection, traditional image processing–based bone suppression, and fully automated pediatric bone suppression. In distance transform–based bone‐edge detection, bone edges were automatically detected by predicting bone‐edge distance‐transform images, which were then used as inputs in traditional image processing. In this processing, pediatric CXR images were labeled by obtaining bone images through a series of traditional image processing techniques. Finally, the pediatric bone‐suppression network was fine‐tuned using the labeled pediatric CXR images. This network was initially pretrained on a public adult dataset comprising 240 adult CXR images (A240) and then fine‐tuned and validated on 40 pediatric CXR images (P260_40labeled) from our customized dataset (named P260) through five‐fold cross‐validation; finally, the network was tested on 220 pediatric CXR images (P260_220unlabeled dataset).

**Results:**

The distance transform–based bone‐edge detection network achieved a mean boundary distance of 1.029. Moreover, the traditional image processing–based bone‐suppression model obtained bone images exhibiting a relative Weber contrast of 93.0%. Finally, the fully automated pediatric bone‐suppression network achieved a relative mean absolute error of 3.38%, a peak signal‐to‐noise ratio of 35.5 dB, a structural similarity index measure of 98.1%, and a bone‐suppression ratio of 90.1% on P260_40labeled.

**Conclusions:**

The proposed fully automated pediatric bone‐suppression network, together with the proposed distance transform–based bone‐edge detection network, can automatically acquire bone and soft‐tissue images solely from CXR images for pediatric patients and has the potential to help diagnose pneumonia in children.

## 1 INTRODUCTION

Pneumonia is a major contributor to morbidity among children globally, particularly in developing nations.^1^ Chest X‐ray [CXR (chest radiography)] is the main tool employed for diagnosing pneumonia.^2^ Although CXR examinations are cost‐effective and convenient, this type of imaging is inherently a form of planar projection and thus leads to an unavoidable overlap of soft tissue and bone in images. Shah et al.^3^ reported that 82%–95% of missed diagnoses in routine CXR examinations result from occlusions caused by a rib or clavicle. Bone‐suppression approaches can be employed to separate CXR images into images showing soft tissue and bone separately, thus helping radiologists or computer‐aided diagnosis systems in their diagnosis process.^4^, ^5^, ^6^, ^7^, ^8^

**FIGURE 1:**
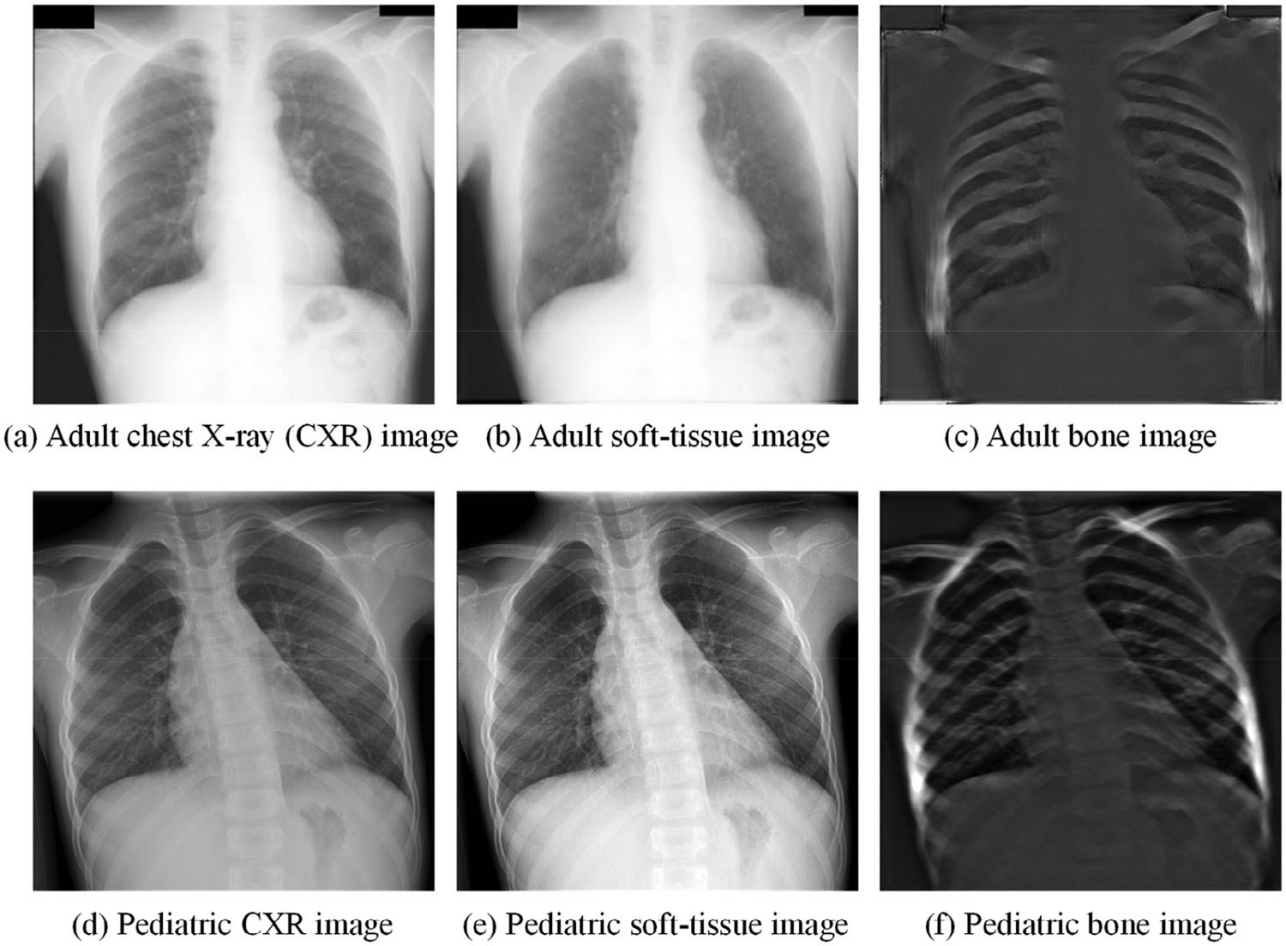
Results of the deep learning–based bone‐suppression network of Suzuki et al.^22^ for adult and pediatric CXR images.

The dual‐energy subtraction (DES)^9^, ^10^, ^11^, ^12^ bone‐suppression approach acquires the projection of data through two energy exposures to derive separate soft‐tissue and bone images. DES is infrequently employed in pediatric settings, and it requires specialized equipment, such as a dual layer or spectral detector. The first proposed bone‐suppression models heavily relied on traditional image processing techniques^13^, ^14^, ^15^, ^16^, ^17^, ^18^, ^19^ and were often framed as a blind source separation (BSS) problem.^20^ When bone suppression is considered a BSS problem, CXR images are assumed to have been generated from multiple independent sources, and the problem is often solved through principal component analysis (PCA).^21^ PCA‐based bone‐suppression models reconstruct bone images by eliminating minor variance information in profiles perpendicular to bone because bone is considered the primary source of variance information in these profiles. Profile sampling is a pivotal step in the application of PCA‐based bone‐suppression models. Existing profile‐sampling approaches—whether based on upper bone edges,^15^ lower bone edges,^16^ or bone centerlines^17^—yield nonlinear bone edges within profiles, and this nonlinearity results in suboptimal bone‐suppression outcomes in bone‐edge regions. Moreover, although traditional image processing–based models can achieve bone suppression without the need for labeled CXR images (i.e., bone and soft‐tissue images), human expertise is required to tune the hyperparameters of these models, making automation challenging. With the widespread integration of deep learning in computer‐aided diagnosis, deep learning–based networks^22^, ^23^, ^24^, ^25^, ^26^, ^27^, ^28^ have emerged as the primary bone‐suppression approaches. Deep learning–based networks used for bone suppression are primarily image‐to‐image networks that learn the mapping relationship between CXR images and soft‐tissue or bone images. Suzuki et al.^22^ proposed a multiresolution massive training artificial neural network for producing bone images. Zarshenas et al.^24^ introduced a bone‐suppression network that considers multiple anatomical structures and frequencies. Eslami et al.^25^ proposed a multitask bone‐suppression network. Yang et al. explored bone‐suppression networks that are cascaded in the gradient domain^23^ or wavelet domain.^28^ However, the deep learning–based networks reviewed in this section require a massive number of labeled CXR images. DES examinations are seldom performed on pediatric patients in clinical practice, making it difficult to collect pediatric DES data. Most existing deep learning–based bone‐suppression networks are applicable to adult patients only; little research has been conducted on pediatric patients. As displayed in Figure 1, the deep learning–based bone‐suppression network of Suzuki et al.,^22^ which is trained on data from adults, cannot effectively eliminate bone occlusion in pediatric CXR images. Therefore, the present study developed a fully automated bone‐suppression network tailored to pediatric patients to improve CXR‐based diagnoses of pediatric pneumonia.

**FIGURE 2:**
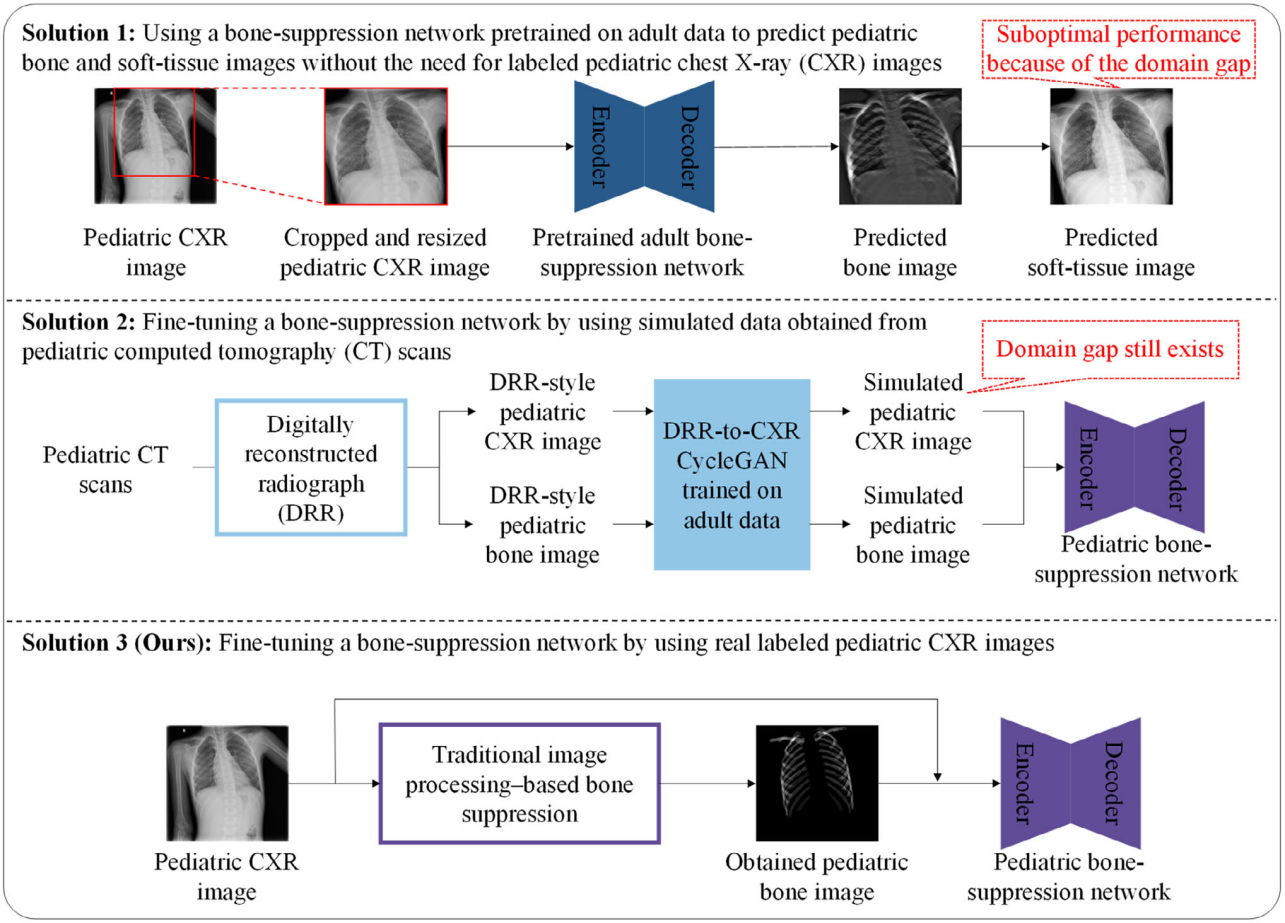
Comparison of our fully automated pediatric bone‐suppression solution and two other solutions.

A comparison of three fully automated pediatric bone‐suppression network solutions is shown in Figure 2. The most straightforward solution, namely solution 1, involves using a bone‐suppression network pretrained on adult data to predict pediatric bone images without the need for labeled pediatric CXR images. Even when CXR images are cropped and resized, pediatric CXR images differ considerably from adult CXR images because pediatric CXR images are often captured with the patient in a nonstandard position because of poor patient compliance. In addition, compared with adult patients, pediatric patients are given a lower dose because of their thinner chest wall and higher radio sensitivity; this lower dose results in clear domain gaps. Unsatisfactory results are obtained with solution 1, possibly because of the domain gap between pediatric and adult CXR images.^29^ Solution 2 involves fine‐tuning a bone‐suppression network by using data obtained from children. In this solution, digitally reconstructed radiograph (DRR)‐style pediatric CXR images and bone images obtained through pediatric computed tomography (CT) scans are employed in a DRR‐to‐CXR CycleGAN.^30^ Through a nonpaired DRR‐to‐CXR CycleGAN image translation network,^31^ the DRR‐style pediatric CXR images and bone images are then translated into simulated pediatric CXR images and simulated pediatric bone images, which serve as data for training a bone‐suppression network. However, the domain gap between simulated CXR images and real CXR images still exists. By contrast, in our approach (solution 3), real labeled pediatric CXR images are acquired to train a bone‐suppression network. Our approach leverages multiple traditional image processing techniques, thus offering higher flexibility and explainability than do other approaches. In the present study, we employed transfer learning^32^, ^33^ to fine‐tune a pediatric bone‐suppression network; this approach is more data‐efficient than is training the model from scratch and thus reduces the number of required labeled CXR images. However, the traditional image processing–based bone‐suppression approach still faces the drawbacks of being labor‐intensive and exhibiting suboptimal bone‐suppression performance.

This paper proposes an efficient labeling approach for fine‐tuning a CXR bone‐suppression network such that the network is applicable to pediatric patients. Our method involves three steps: distance transform–based bone‐edge detection, traditional image processing–based bone suppression, and fully automated bone suppression. In the first step, the bone edges are automatically detected for use in the next step, effectively reducing labor costs. In the second step, multiple traditional image processing techniques are combined to obtain high‐quality pediatric bone images, thereby achieving the labeling of pediatric CXR images. In the third step, the pediatric bone‐suppression network is trained on the labeled pediatric CXR images. The effectiveness of each step was evaluated on a customized labeled dataset (named P260_40labeled). The final network was then applied to an open large‐scale pediatric CXR dataset (VinDr‐PCXR) for thoracic diseases, and the results demonstrated that our fully automated bone‐suppression network can effectively improve the accuracy of computer‐aided diagnostic classification networks for pediatric patients.

## 2 METHODS

**FIGURE 3:**
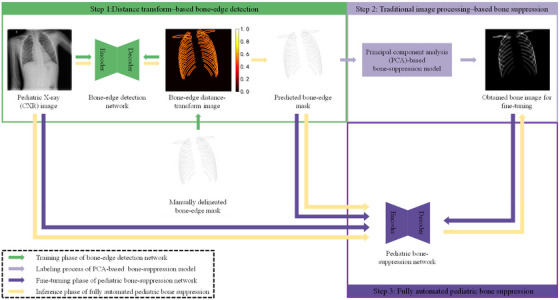
Proposed labeling and fine‐tuning method for a fully automated bone‐suppression network applicable to pediatric CXR images.

Figure 3 illustrates the overall process of the developed fully automated bone‐suppression network, which performs three steps: distance transform–based bone‐edge detection, traditional image processing–based bone suppression, and fully automated pediatric bone suppression.

### 2.1 Distance transform–based bone‐edge detection

Traditional image processing–based bone‐suppression models usually rely on bone edges for guidance. Although manually delineating bone edges results in high accuracy, this process is labor‐intensive. Moreover, manually delineated bone edges introduce uncertainty, which influences the quality of the data used to train the segmentation network and thus negatively affects segmentation performance. To address this uncertainty, we formulated bone‐edge detection as a regression problem. We trained a bone‐edge detection network to learn the correlation between pediatric CXR images and bone‐edge distance‐transform images. In this paper, the bone‐edge detection network, pediatric CXR image, manually delineated bone‐edge mask, and bone‐edge distance‐transform image are denoted by F_e_, **I**, **E**_m_, and **E**_dt_, respectively. The process of predicting **E**_dt_ can be expressed as follows:

(1) $${\hat{E}}_{\mathrm{dt}}=F_{e}\left(I\right).$$

The ground truth of the bone‐edge distance‐transform image is acquired from **E**_dt_ = Tr_dis_(**E**_m_), where Tr_dis_ represents the distance‐transform operator, which is precisely calculated using the following equation:

(2) $$t_{ij}=e^{\frac{-\mathrm{Min}(\mathrm{Dis}\left(m_{ij},\;1\right))}{\left|\sigma \right|}},$$

where 1 is the set of positive pixels in **E**_m_; σ is an adjustable factor that controls the degree of softening; and t*_ij_* and m*_ij_* are the pixels of **E**_dt_ and **E**_m_, respectively. Equation (2) indicates that a distance between m*_ij_* and 1 of 0 and +∞ results in the corresponding t*_ij_* value being 1 and 0, respectively. The operator Tr_dis_ softens the binary mask **E**_m_ into **E**_dt_, whose pixel values range from 0 to 1; this step decreases the uncertainties associated with the manual delineation of bone edges. Smaller |σ| values result in less softening and greater preservation of the resemblance of **E**_dt_ to **E**_m_. Ultimately, the problem of distance transform–based bone‐edge detection is translated into a problem involving the minimization of the mean squared error between **E**_dt_ and $\hat{E_{\mathrm{dt}}}$ to derive F_e_. For comparison, the bone‐edge segmentation network is trained using the binary cross‐entropy loss function to minimize the disparity between the predicted bone‐edge masks and their ground truth. Both the detection and segmentation networks can be used to predict the bone‐edge masks by adopting an intensity threshold and conducting skeleton extraction on the output logits. From the bone‐edge distance‐transform images, bone edges with a width of approximately 3 can be obtained when a threshold of 0.8 is used, which is beneficial for obtaining bone edges with favorable continuity and accuracy. Therefore, a fixed value of 0.8 is used as the threshold. To process anterior ribs, posterior ribs, and clavicles separately, three networks dedicated to the three specific types of bone are trained. The bone‐edge masks predicted by the three networks are then processed through dot multiplication to obtain the final predicted bone‐edge mask. The implementation details are introduced in Section 2.6.

### 2.2 Traditional image processing–based bone suppression

**FIGURE 4:**
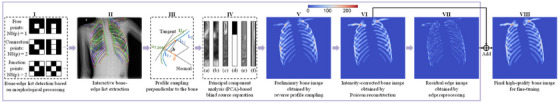
Detailed process of the PCA‐based bone‐suppression model. (I) Bone‐edge lists obtained through morphological processing of the binary bone‐edge mask. (II) The bone‐edge list is divided into 32 anterior rib edges, 44 posterior rib edges, and 4 clavicle edges through a semiautomatic process. (III) Sampling is performed perpendicular to the bone to produce elongated structures, as shown in the profile image presented in (IV.a). Subtraction of the estimated profile background image (IV.b) from the (IV.a) yields the profile foreground images shown in (IV.c), where bone structure serves as the primary information. Subsequently, (IV.d) profile maps are obtained through k‐means clustering, with corrupted profile parts highlighted (depicted in black and crossed by other structures, such as other bone and a catheter). Next, the (IV.e) preliminary bone profile images are reconstructed by conducting PCA on the uncorrupted profiles (shown in white). The (IV.f) bone profile images are derived by performing smoothing along the bone edges. Finally, the (IV.f) bone profile images are subjected to reverse profile sampling to obtain preliminary bone images (V). (VI) Corrected bone image obtained through Poisson reconstruction of (V). (VII) Residual edge image obtained through edge reprocessing of preliminary soft‐tissue image derived by subtracting (VI) from the CXR image. (VIII) Final high‐quality bone image obtained through adding (VII) to (VI).

Figure 4 shows the detailed processing flow of the PCA‐based bone‐suppression model, which uses multiple traditional image processing techniques.

#### 2.2.1 Detection and extraction of bone‐edge lists

The predicted bone‐edge masks had to be postprocessed into bone‐edge lists for profile sampling. Morphological processing^34^ was employed for bone‐edge list detection (Figure 4I). Initially, erosion operations were employed to obtain bone‐edge skeletons. On the basis of eight‐neighborhood information, each pixel in the image of the bone‐edge skeleton was identified as a free, connection, or junction point. Thus, all bone‐edge lists were detected from the binary bone‐edge masks. Eventually, these bone‐edge lists were interactively connected if they belonged to the same bone edge (Figure 4II). We extracted 32 anterior rib edges, 44 posterior rib edges, and 4 clavicle edges from each bone‐edge mask. These bone‐edge lists, which encompassed both upper and lower bone edges, can be represented as follows:

(3) $$\mathrm{Bone}\;\mathrm{edge}\;\mathrm{lists}=\left(\left(U_{1},L_{1}\right),\left(U_{2},L_{2}\right),\text{…}\left(U_{80},L_{80}\right)\right),$$

(4) $$U_{1}=\left(u_{11},u_{12}\text{…},u_{1n}\right),$$

where U_1_ and L_1_ are the upper and lower edges of the first bone, respectively; u_11_ and u_12_ are the first and second points of U_1_, respectively; and *n* is the length of U_1_.

#### 2.2.2 Profile sampling

Profile sampling (Figure 4III) was employed to straighten the bone edges and create conditions for PCA‐based BSS. We proposed a profile‐sampling technique based on the upper and lower edges to render the upper and lower bone edges as straight lines in the profile image (Figure 4IV.a). Figure 4 illustrates the profile sampling at u_7,200_ as an example. Initially, we established the tangent and normal lines at u_7,200_ and then searched for the intersection point (l_7,*_) of L_7_ and the normal line. This search was completed as follows:

(5) $$\begin{array}{ccc} l_{7,*} & = & \underset{j}{\mathrm{argmin}}\left|\left|\vec{e},\vec{u_{7,200}l_{7,j}}\right|/\left|\vec{e}\right|\right|\\ & = & \underset{j}{\mathrm{argmin}}h,\;s.t.\left|\vec{u_{7,200}l_{7,j}}\right|<100, \end{array}$$

where $\vec{e}$ is the unit vector in the normal direction. The determinant value for vectors $\vec{e}$ and $\vec{u_{7,200}l_{7,j}}$—that is $|\vec{e},\vec{u_{7,200}l_{7,j}}|$—is equal to the area of the parallelogram in Figure 4(III). Thus, |e⃗,u7,200l7,j⃗|/|e⃗,u7,200l7,j⃗||e⃗||e⃗| represents the height (*h*) of the parallelogram. According to Equation (5), each point on U_7_ and its corresponding l_7,*_ point on L_7_ can help determine the direction of profile sampling. A two‐point linear equation can be used to sample a fixed number of pixels on and outside the line connecting points u_7,200_ and l_7,*_ to transform bone structures into a profile image. We sampled 10 and 40 pixels on the outer and inner sides, respectively, for each anterior rib, posterior rib, and clavicle.

#### 2.2.3 PCA‐Based BSS

PCA‐based BSS involves four steps: background filtering, profile clustering, PCA, and bone‐edge‐direction smoothing.

The aim of background filtering is to eliminate low‐frequency background information, a process that contributes to the performance of PCA. We approximated low‐frequency profile background images (Figure 4IV.b) by using the spline‐smoothing algorithm,^35^, ^36^ which was configured with a large‐scale smoothing kernel having a size of 1000 pixels and with three pixels on both sides of the profiles. Subtracting the approximated images from the profile images yielded the profile foreground images (Figure 4IV.c).

In profile clustering, the k‐means algorithm^37^ was employed to categorize profiles into two groups—uncorrupted profiles (depicted in white in Figure 4IV.d), which formed the majority group, and corrupted profiles (marked in black in Figure 4IV.d, which are crossed by other structures, such as other bones and catheters), which formed the minority group. To minimize the influence of corrupted profiles on the results, we substituted these profiles with the mean value of the uncorrupted profiles.

PCA is an analytical method for performing BSS, which facilitates bone suppression in profile images. The equations related to PCA are expressed as follows:

(6) P=ZW,

(7) $$P_{B}=Z_{r}W_{r},$$

where **P** represents the profile images obtained after background filtering and profile clustering. Moreover, **W** represents the mixing matrix of various sources (**Z**). For dimension reduction in PCA, the principal components are considered as **Z**, and **Z** *_r_* and its corresponding mixing matrix **W** *_r_* are extracted by retaining the *r* principal components that have the greatest cumulative contribution to the variance. In this study, preliminary bone profile images (Figure 4IV.e) were estimated using Equation (7), and *r* was obtained on the basis of a cumulative variance of 95%; thus, important information was retained in **P_B_**.

Bone‐edge‐direction smoothing was subsequently performed to eliminate soft‐tissue from the preliminary bone profile images. Smoothing operations were then employed along the bone‐edge direction, with the kernel size set as 30. These operations yielded the final bone profile image (Figure 4IV.f), which is denoted as **P_B_**.

#### 2.2.4 Reverse profile sampling

Reverse profile sampling involved mapping **P_B_** back to the image space by using the positional correspondence of the profile sampling. The unsampled pixels were filled in through nearest‐neighbor interpolation to obtain preliminary bone image $I_{B}^{0}$ (Figure 4V) in the image space.

#### 2.2.5 Poisson reconstruction

The background of $I_{B}^{0}$ was often not 0 because the background filtering process did not ensure that the background intensity of **P** was consistently 0, which resulted in excessive suppression. To rectify the background intensity of $I_{B}^{0}$, we employed the Poisson fusion algorithm,^38^, ^39^ which identifies regions with a gradient and intensity of 0 as nonbone regions and conducts integration to reconstruct corrected bone image $I_{B}^{1}$ (Figure 4VI) on the basis of the gradient information. Bone region masks were obtained using a 3 × 3 kernel to dilate the bone mask obtained based on bone edges, whereas nonbone region masks encompassed the region outside the bone region masks.

#### 2.2.6 Edge reprocessing

Although the bone‐edge‐direction smoothing process effectively eliminated soft‐tissue from **P_B_**, it possibly reduced the suppression performance in the bone‐edge regions, leaving residual bone edges. Therefore, we reused PCA‐based BSS method to eliminated these bone edges in preliminary soft‐tissue image derived by subtracting $I_{B}^{1}$ from **I**. In contrast to the processing of bone, profile sampling for edge reprocessing relied on centerlines. Ten pixels were sampled on each side, with the middle 10 pixels considered as the edge and the outer 5 pixels considered as the background. A smaller kernel was employed in the bone‐edge‐direction smoothing process used for edge reprocessing than in bone processing. The extracted residual edge image (Figure 4VII) was added to the corrected bone image ($I_{B}^{1}$) obtained through Poisson reconstruction to obtain the final high‐quality bone image **I_B_** (Figure 4VIII). The final soft‐tissue image **I_S_** was derived by subtracting **I_B_** from **I**. All traditional image processing–based bone‐suppression techniques were executed using MATLAB (MathWorks, Natick, MA, USA).

### 2.3 Fully automated pediatric bone suppression

To obtain the fully automated pediatric bone‐suppression network, a multiscale image‐to‐image network was trained on labeled pediatric CXR images to avoid interactive bone‐edge list extraction. As revealed by Yang et al.,^23^ gradient information is crucial for training bone‐suppression networks. In addition, bone edges are necessary input information for the traditional image processing–based bone‐suppression models used to label pediatric CXR images. The input layer of the developed fully automated pediatric bone‐suppression network integrated intensity‐domain, gradient‐domain, and bone‐edge information. Original CXR images, images of the X‐direction gradient, images of the Y‐direction gradient, and predicted bone‐edge masks were concatenated as the input channels of the pediatric bone‐suppression network. The gradient images were obtained using Sobel filters in the OpenCV Python package. Most pixels in **I_B_** had a value of 0, and the data distribution in **I_B_** was simpler than that in **I_S_**; thus, the task of the output layer, which had to predict **I_B_**, was simplified. Transfer learning was used to alleviate the problem of insufficient labeled pediatric CXR images. The pediatric bone‐suppression network was first pretrained on the A240 dataset and then fine‐tuned on the P260_40labeled dataset. The fine‐tuned pediatric bone‐suppression network, together with the trained bone‐edge detection network, can achieve fully automated bone suppression in CXR images for pediatric patients. The implementation details are introduced in Section 2.6.

To validate the effectiveness of concatenating bone‐edge channels and the pretraining process, several networks with different configurations were developed. ABSN and PBSN refer to bone‐suppression networks for adult and pediatric patients, and these networks were trained from scratch on the A240 and P260_40labeled datasets, respectively. PBSN‐E is a version of PBSN that concatenates the bone‐edge masks predicted by the bone‐edge detection networks and then uses the concatenation results as input for the bone‐suppression network. PBSN‐P refers to a network pretrained on A240 and then fine‐tuned on P260_40labeled. Moreover, PBSN‐E‐P refers to the combination of PBSN‐E and PBSN‐P.

### 2.4 Datasets

This study employed data from three sources, namely our collected pediatric CXR dataset (P260), a publicly available dataset for adult bone suppression (A240^40^), and an open large‐scale pediatric CXR dataset (VinDr‐PCXR^41^) on thoracic diseases.

#### 2.4.1 P260

The P260 dataset contains 260 pediatric CXR images from patients aged < 12 years (the cut‐off point for transition of care) who visited the Zhujiang Hospital of Southern Medical University (Guangzhou, Guangdong Province, China). All CXR images were obtained between January 1 and April 28, 2019, and they have a size ranging from 1021 × 1085 to 2208 × 2432 pixels and were saved as 16‐bit DICOM files. Forty of these CXR images were selected using stratified sampling across diverse age groups. To train the developed bone‐edge detection network, we invited an expert radiologist to manually delineate the edges of the anterior ribs, posterior ribs, and clavicles in the 40 selected pediatric CXR images; this delineation was conducted using RadiAnt DICOM Viewer (Medixant, Poznan, Poland). MATLAB (MathWorks) was employed to obtain bone‐edge masks. We obtained the pediatric bone images of these 40 selected pediatric CXR images by using our PCA‐based bone‐suppression model. The 40 labeled pediatric CXR images (P260_40labeled) were employed for five‐fold cross‐validation of the bone‐edge detection network and pediatric bone‐suppression network. To prevent data leakage, the partitioning of each fold for the pediatric bone‐suppression network was aligned with that for the bone‐edge detection network. The remaining 220 unlabeled pediatric CXR images (P260_220unlabeled) were used to test the generalizability of the pediatric bone‐suppression network. Table 1 shows the age and sex distribution of the patients whose CXR images are included in the P260 dataset.

**TABLE 1:** Age and sex distribution of the patients whose CXR images are included in the P260 dataset.

	P260_40labeled Fold 1	P260_40labeled Fold 2	P260_40labeled Fold 3	P260_40labeled Fold 4	P260_40labeled Fold 5	P260_220unlabeled
0–1 years old	0	0	0	0	0	6
1–2 years old	1	1	0	0	0	3
2–3 years old	0	0	1	0	0	0
3–4 years old	0	0	0	1	1	5
4–5 years old	2	1	0	0	0	16
5–6 years old	0	0	1	1	1	31
6–7 years old	2	1	1	0	0	41
7–8 years old	0	2	2	3	2	28
8–9 years old	1	1	1	1	1	28
9–10 years old	1	1	1	1	1	26
10–11 years old	1	1	0	0	1	16
11–12 years old	0	0	1	1	1	20
Male	7	6	4	6	7	144
Female	1	2	4	2	1	76
Total	8	8	8	8	8	220

#### 2.4.2 A240

The A240 dataset consists of 240 pairs of adult CXR images and soft‐tissue images in the PNG format; these images have a size of 1024 × 1024 pixels. The original CXR images were digitized using an LD‐4500 or LD‐5500 laser film digitizer (Konica, Tokyo, Japan). A240 was mainly used for pretraining to obtain parameters for initializing the pediatric bone‐suppression network. The five‐fold cross‐validation data for ABSN were obtained through random sampling.

#### 2.4.3 VinDr‐PCXR

The VinDr‐PCXR dataset is in the DICOM format, and its data were retrospectively collected from three major hospitals in Vietnam and cover the period 2020 to 2021. Each image, which ranges in size from 771 × 788 to 3072 × 3000 pixels, was meticulously annotated for 15 diseases by an expert radiologist. To mitigate class imbalance, we reduced the dataset size by including only six disease categories. We employed the training and validation sets of the original VinDr‐PCXR dataset instead of cross‐validation for comparison with other studies. Specifically, the training and validation subsets encompassed 7645 and 1386 images, respectively. The distribution of disease labels within the training set was as follows: no finding, 5143 images; bronchitis, 842 images; bronchopneumonia, 545 images; other infection, 412 images; bronchiolitis, 497 images; and pneumonia, 392 images. In the validation set, the corresponding counts were 907, 174, 84, 77, 90, and 89 images, respectively. To demonstrate that the proposed PBSN‐E‐P network can effectively improve the performance of computer‐aided diagnosis classification networks for pediatric CXR images, VinDr‐PCXR was processed by ABSN and PBSN‐E‐P to obtain VinDr‐PCXR_ABSN_ and VinDr‐PCXR_PBSN‐E‐P_, respectively. We compared the performance of multilabel classification networks (based on ResNet50) on VinDr‐PCXR, VinDr‐PCXR_ABSN_, and VinDr‐PCXR_PBSN‐E‐P_.

### 2.5 Evaluation metrics

Inspired by research on bone‐edge segmentation in CXR images,^42^ we employed recall, precision, dice (equal to the F1 score), and mean boundary distance (MBD)^42^, ^43^ to assess the bone‐edge detection outcomes. MBD is used specifically to measure edge distance and is calculated as follows:

(8) $$\mathrm{MBD}\left(S,T\right)=\frac{1}{2}\left(\sum_{i}d\left(s_{i},T\right)/\left|\left\{s_{i}\right\}\right|+\sum_{j}d\left(t_{j},S\right)/\left|\left\{t_{j}\right\}\right|\right),$$

(9) $$d\left(s_{i},T\right)=\underset{j}{\mathrm{Min}}(s_{i}-t_{j}),$$

where s*_i_* and t*_i_* are the positive pixels in the source binary image **S** and target binary image **T**, respectively; |{s*_i_*}| and |{t*_i_*}| represent the number of positive pixels in **S** and **T**, respectively; and d(s*_i_*, **T**) is the minimum Euclidean distance from s*_i_* to {t*_i_*}. MBD is measured in pixels, and a smaller MBD indicates the existence of less edge deviation between **S** and **T**.

The relative Weber contrast (RWC)^17^ was employed to assess the performance of traditional image processing–based bone‐suppression models. The RWC can be used to determine bone‐suppression quality by comparing the contrast between the bone region and the soft‐tissue region. The equation for its calculation is as follows:

(10) $$\mathrm{WC}\left(I\right)=\frac{\mathrm{Mean}\left(I,\Omega_{B}\right)-\mathrm{Mean}\left(I,\Omega_{S}\right)}{\mathrm{Mean}\left(I,\Omega_{S}\right)},$$

(11) $$\mathrm{RWC}=\frac{\mathrm{WC}\left(I\right)-\mathrm{WC}\left(I_{S}\right)}{\mathrm{WC}\left(I\right)},$$

where Mean(**I**, **Ω_B_**) and Mean(**I**, **Ω_S_**) are the average intensities of the CXR images (denoted as **I**) in the bone region (denoted as **Ω_B_**) and soft‐tissue region (denoted as **Ω_S_**), respectively. The parameter **Ω_B_** was obtained by conducting the erosion operation with the 3 × 3 kernel on the bone mask obtained through an 50% intensity threshold of the predicted bone image, whereas **Ω_S_** was obtained by subtracting the dilated bone‐segmentation results from the results of an off‐the‐shelf lung‐field segmentation network.^44^ The RWC was derived from the Weber contrast of **I** and **I_S_**, as shown in Equation (11). In an ideal scenario, WC(**I**) is greater than 0, WC(**I_S_**) is equal to 0, and the RWC equals 1. However, in practical situations, the RWC typically ranges from 0 to 1 and may even surpass 1, which would indicate excessive bone suppression.

To evaluate our fine‐tuned pediatric bone‐suppression network, we used four metrics: the peak signal‐to‐noise ratio (PSNR),^23^ structural similarity index measure (SSIM),^45^ relative mean absolute error (RMAE),^23^ and bone‐suppression ratio (BSR).^17^ The PSNR and SSIM are metrics commonly used to evaluate denoising, and high values of these metrics indicate high bone‐suppression performance. We used the default settings of the scikit‐image library function to calculate PSNR‐S and SSIM‐S for the predicted soft‐tissue image and its ground truth within **Ω_B_**. The RMAE values for the predicted bone image and its ground truth were calculated using the following equation:

(12) $$\begin{array}{ccc} \mathrm{RMAE}-B & = & \sqrt{\frac{1}{N}\sum_{\left(x,y\right)\in \Omega_{B}}\left|I_{B}\left(x,y\right)-\hat{I_{B}}\left(x,y\right)\right|}/\left(\mathrm{Max}\left(I_{B},\Omega_{B}\right)\right.\\ & & \left.-\;\mathrm{Min}\left(I_{B},\Omega_{B}\right)\right). \end{array}$$

where *N* refers to the number of positive pixels in **Ω_B_**; (*x*, *y*) represents the coordinates of the positive pixels; and Max(**I_B_**, **Ω_B_**) and Min(**I_B_**, **Ω_B_**) are the maximum and minimum values of **I_B_** in **Ω_B_**, respectively. A smaller RMAE value indicates higher bone‐suppression quality. The equation for calculating the BSR is as follows:

(13) $$\mathrm{BSR}=1-\sum_{\left(x,y\right)\in \Omega_{B}}{\left(I_{S}\left(x,y\right)-\hat{I_{S}}\left(x,y\right)\right)}^{2}/\sum_{\left(x,y\right)\in \Omega_{B}}I_{B}{\left(x,y\right)}^{2}.$$

A BSR of 1 indicates perfect performance.

The following metrics, summarized by a previous study,^46^ were utilized to evaluate the performance of multilabel classification for the VinDr‐PCXR dataset: four label‐based metrics, namely micro F1 score, macro F1 score, weighted F1 score, and sample F1 score, and five example‐based metrics, namely subset accuracy, accuracy_exam_, F1 score_exam_, one‐error, and coverage.

### 2.6 Implementation details

To perform distance transform–based bone‐edge detection, the sizes of images were standardized to 512 × 512 pixels, and the images were preprocessed through an edge‐preserving smoothing operator called the guided filter.^47^ The guided filter controls the filtering effect through two parameters: window size and regularization factor. A larger window size allows the filter to search for local consistency within a larger area, which facilitates the smoothing of noise and edges. A lower regularization factor causes the output image to be closer to the input image but might result in the introduction of noise. We adopted a large window of 90 × 90 pixels and a small regularization factor of 10^8^ to eliminate low‐frequency information. The data augmentation procedure used in this study encompassed random rotation, translation, flipping, and normalization; the softening factor σ was set at 5. We employed the Tiramisu network^48^ with the Adam optimizer, and our settings included a strategy of cosine annealing warm restarts (implemented using the Torch package with the parameters T_0 = 10 and T_mult = 2) with an initial learning rate of 0.0001, a batch size of 6, and training over 300 epochs. Distance transform–based bone‐edge detection was implemented using Python 3.9 and PyTorch 2.0.1.

For fully automated pediatric bone suppression, images were resized to 512 × 512 pixels, and the same data augmentation, network settings, optimizer, and learning rate strategy were employed as for the bone‐edge detection. The batch size was set as 6. PBSN‐E‐P was pretrained for 300 epochs on A240 and then fine‐tuned for 300 epochs on P260_40labeled. Python 3.9 and PyTorch 2.0.1 were utilized for the pretraining and fine‐tuning.

The computer used for training the deep learning network was equipped with two A30 graphics processing units with 24 GB of memory.

## 3 RESULTS

### 3.1 Distance transform–based bone‐edge detection network versus bone‐edge segmentation network

Table 2 summarizes the five‐fold cross‐validation performance of the distance transform–based bone‐edge detection and bone‐edge segmentation networks. The results indicate that our distance transform–based bone‐edge detection network outperformed the bone‐edge segmentation network on four metrics: MBD, recall, precision, and dice (Table 2). The recall and dice (F1 score) of our distance transform–based bone‐edge detection were significantly more favorable than those of the bone‐edge segmentation network. The recall, precision, and dice (F1 score) results were calculated by comparing the manually delineated bone edges with the predicted bone edges obtained by adopting an intensity threshold and conducting skeleton extraction on the output logits. Because the manually delineated bone edges were uncertain, the relatively small values for the recall, precision, and dice were reasonable.

**TABLE 2:** Five‐fold cross‐validation performance of the developed distance transform–Based bone‐edge detection network and bone‐edge segmentation network on P260_40labeled.

Networks	MBD	Recall (%)	Precision (%)	Dice (F1‐score, %)
Bone‐edge segmentation	1.206 ± 0.473	43.7 ± 4.8	44.7 ± 4.4	44.1 ± 4.1
Distance‐transform–based bone‐edge detection (Ours)	1.029 ± 0.355	50.0 ± 5.4^*^	45.9 ± 5.1	47.7 ± 4.6^*^

* *p* ≤ 0.05.

### 3.2 Ablation study on the traditional image processing–based bone‐suppression model

**FIGURE 5:**
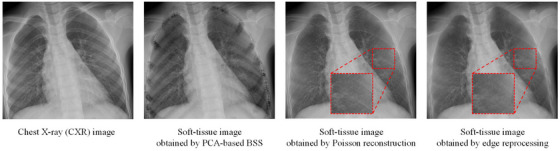
Bone‐suppression results at different process nodes of the PCA‐based bone‐suppression model. The red dashed line displays an enlarged partial view of the lungs.

Figure 5 illustrates the improvement in bone‐suppression performance resulting from Poisson reconstruction and edge reprocessing. The quantitative analysis provided in Table 3 underscores the efficacy of the background intensity correction performed through Poisson reconstruction. The RWC for PCA‐based BSS exceeded 100%, indicating excessive bone suppression. Poisson reconstruction was used to correct the background intensity of bone images, which resulted in an RWC of 85.4%. Edge reprocessing further improved the RWC by 7.6% to 93.0%. Table 3 presents a comparison of the bone‐suppression performance of the PCA‐based bone‐suppression model when it used manually delineated bone edges (PCA‐M) and detected bone edges (PCA‐D). Although the RWC of PCA‐D was lower than that of PCA‐M, PCA‐D reduced labor costs and preserved the possibility of fully automated pediatric bone suppression.

**TABLE 3:** Bone‐suppression performance at various process nodes of the PCA‐based bone‐suppression model for P260_40labeled when manually delineated bone edges (PCA‐M) or detected bone edges (PCA‐D) were employed.

Process nodes	RWC of PCA‐based bone‐suppression model using manually delineated bone edges (PCA‐M, %)	RWC of PCA‐based bone‐suppression model using detected bone edges (PCA‐D, %)
PCA‐based BSS	179.7 ± 36.1	148.8 ± 42.0
Poisson reconstruction	85.4 ± 18.1	73.0 ± 18.3
Edge reprocessing	93.0 ± 18.5	83.6 ± 21.4

### 3.3 Effectiveness of concatenating a bone‐edge channel and pretraining

Table 4 presents a comparison of the performance of bone‐suppression networks with different configurations. On the P260_40labeled dataset, PBSN‐E and PBSN‐P outperformed PBSN in terms of the BSR and RMAE‐B, which verified the effectiveness of concatenating a bone‐edge channel and then conducting pretraining. PBSN‐E‐P had the best performance on the P260_40labeled dataset in terms of RMAE‐B, PSNR‐S, SSIM‐S, and BSR, which confirmed the synergistic effect of concatenating a bone‐edge channel and fine‐tuning. On the A240 dataset, PBSN‐E‐P exhibited less performance degradation than did PBSN, PBSN‐E, and PBSN‐P.

**TABLE 4:** Performance of bone‐suppression networks with different configurations on the A240 and P260_40labeled datasets.

Networks	Pretraining	Training	Validation	RMAE‐B (%)	PSNR‐S (dB)	SSIM‐S (%)	BSR (%)
ABSN		A240	P260_40labeled	5.87 ± 0.8	29.1 ± 1.5	97.4 ± 0.8	22.3 ± 36.2
PBSN		cross‐validation on P260_40labeled		3.46 ± 0.43	35.2 ± 1.5	98.1 ± 0.4	89.4 ± 3.6
PBSN‐E		cross‐validation on P260_40labeled		3.46 ± 0.43	35.2 ± 1.6	98.1 ± 0.4	89.5 ± 3.4
PBSN‐P	A240	cross‐validation on P260_40labeled		3.44 ± 0.45	35.3 ± 1.6	98.1 ± 0.4	89.6 ± 3.5
PBSN‐E‐P	A240	cross‐validation on P260_40labeled		3.38 ± 0.43	35.5 ± 1.6	98.1 ± 0.4	90.1 ± 3.4
ABSN		cross‐validation on A240		5.82 ± 2.00	43.9 ± 3.8	99.8 ± 0.1	78.9 ± 7.8
PBSN		P260_40labeled	A240	8.72 ± 3.05	37.5 ± 3.6	99.7 ± 0.2	16.9 ± 5.6
PBSN‐E		P260_40labeled	A240	8.59 ± 2.96	37.6 ± 3.7	99.7 ± 0.2	19.5 ± 7.2
PBSN‐P	A240	P260_40labeled	A240	8.47 ± 2.96	37.8 ± 3.7	99.7 ± 0.2	23.3 ± 8.4
PBSN‐E‐P	A240	P260_40labeled	A240	8.40 ± 2.94	37.9 ± 3.7	99.7 ± 0.2	24.9 ± 9.2

### 3.4 Quantitative and qualitative results of PBSN‐E‐P for various age groups

**FIGURE 6:**
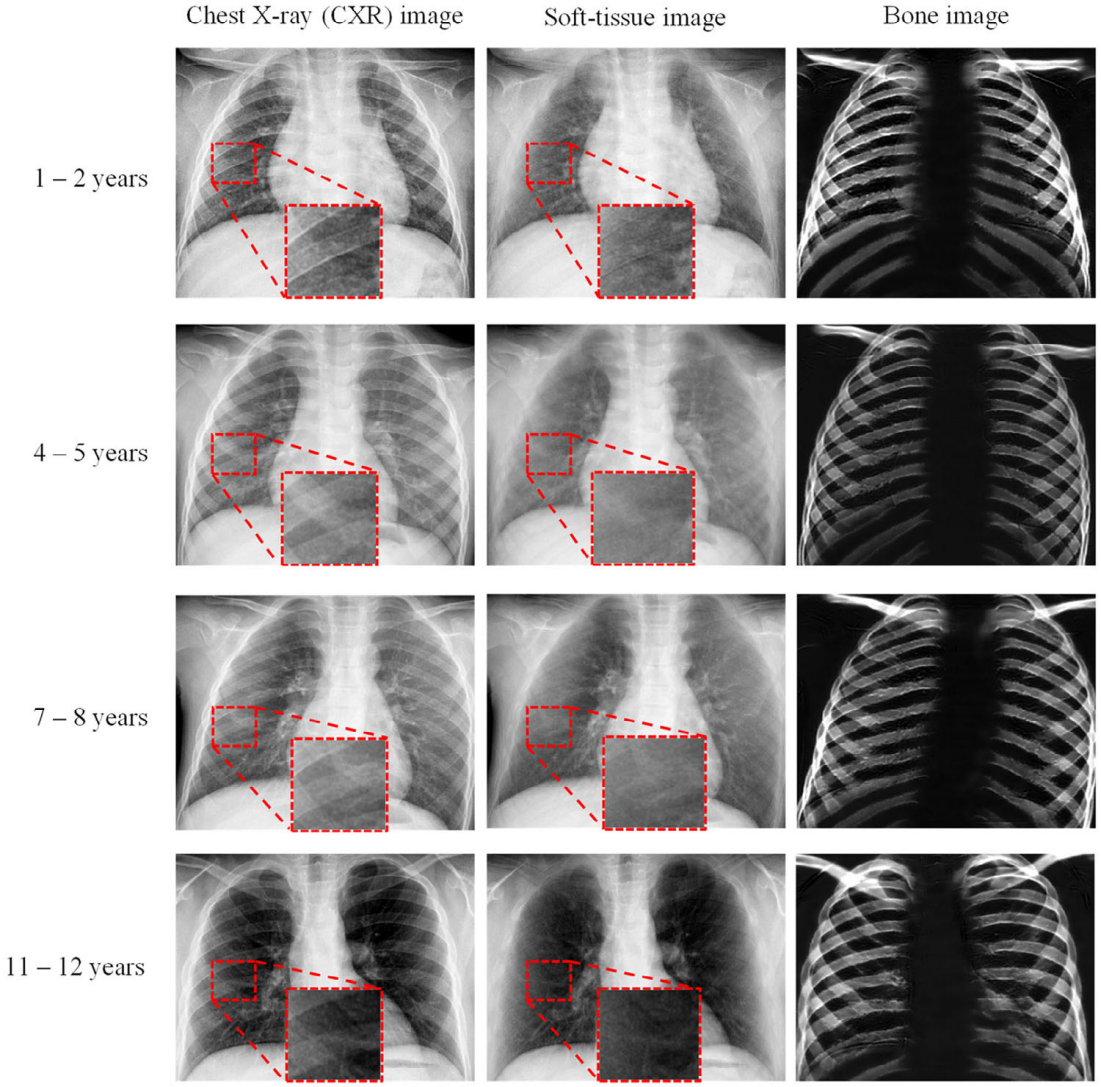
Qualitative results of PBSN‐E‐P on the P260_220unlabeled dataset for various age groups. The area within the red dashed line displays an enlarged partial view.

To assess the generalizability of the developed pediatric bone‐suppression network across age groups, we conducted a comprehensive quantitative and qualitative analysis involving the pediatric CXR images in the P260_220unlabeled dataset. As presented in Table 5, the macro‐average RWC is 84.5%, which indicats commendable bone‐suppression performance for pediatric patients aged less than 12 years. Nonetheless, the performance for the 0–3‐year‐old group is slightly worse, possibly because of data imbalance in the P260_220unlabeled dataset, as revealed in Table 1. Figure 6 shows the observable bone residues within the predicted soft‐tissue images of patients aged 0–3 years. Overall, our pediatric bone‐suppression network exhibited effective CXR bone‐suppression capability for patients aged less than 12 years.

**TABLE 5:** Generalizability of PBSN‐E‐P on the P260_220unlabeled dataset for various age groups.

Age (years)	Number of train sample on P260_40labeled	Number of test sample on P260_220unlabeled	RWC (%)
0–3	3	9	76.9 ± 32.6
3–5	5	21	85.8 ± 22.9
5–7	7	72	86.2 ± 23.3
7–9	14	56	83.9 ± 19.5
9–12	11	62	83.3 ± 16.9
0–12	40	220	84.5 ± 21.2

### 3.5 Comparison of various bone‐suppression approaches

Table 6 presents a comparison of the advantages and disadvantages of various bone‐suppression approaches, including DES, the traditional image processing–based bone‐suppression model, and the deep learning–based bone‐suppression network. DES does not require any labor or computer‐based postprocessing; however, it requires a high radiation dose. ABSN avoids high radiation doses but relies on DES data as training data. Although PCA‐M enables unsupervised bone suppression, 50 min are required for the manual delineation of bone edges in each CXR image. The semiautomated PCA‐D model uses detected bone edges to save the time involved in manually delineating bone edges; however, 5 min are still required for interactive bone‐edge list extraction. Although our fully automated PBSN‐E‐P network has a lower RWC than does PCA‐M, PBSN‐E‐P can achieve fully automated bone suppression in CXR images obtained from pediatric patients.

**TABLE 6:** Multidimensional comparison of various bone‐suppression approaches.

Bone‐suppression approaches	Validation	RWC	Radiation dose	Time for manually delineating bone edges	Time for interactive bone‐edge list extraction	Time for automatic computer post‐processing
DES	A240	41.1 ± 6.4	High	0	0	0
ABSN	A240	43.0 ± 17.4	Low	0	0	0.05 s
PCA‐M	P260_40labeled	93.0 ± 18.5	Low	50 min	0	3 min
PCA‐D	P260_40labeled	83.6 ± 21.4	Low	0	5 min	3 min
PBSN‐E‐P	P260_40labeled	85.5 ± 15.8	Low	0	0	0.05 s

### 3.6 Performance of various bone‐suppression networks on VinDr‐PCXR

**FIGURE 7:**
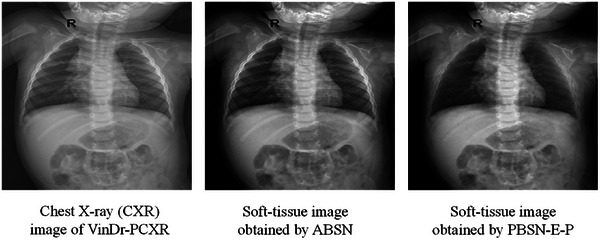
Bone‐suppression results of ABSN and PBSN‐E‐P on VinDr‐PCXR.

The soft‐tissue images of VinDr‐PCXR_ABSN_ and VinDr‐PCXR_PBSN‐E‐P_ (Figure 7) indicate that PBSN‐E‐P has higher generalizability than does ABSN on VinDr‐PCXR. Table 7 details the performance of different multilabel classification networks on VinDr‐PCXR, VinDr‐PCXR_ABSN_, and VinDr‐PCXR_PBSN‐E‐P_. The results obtained on VinDr‐PCXR_PBSN‐E‐P_ exhibited the highest performance for seven metrics but the worst performance in terms of macro F1 score and weighted F1 score. This discrepancy might be attributable to the susceptibility of the macro F1 score and weighted F1 score to class imbalance. Moreover, the low values for these two metrics does not necessarily indicate low classification performance.

**TABLE 7:** Performance of multilabel classification networks on VinDr‐PCXR, VinDr‐PCXR_ABSN_, and VinDr‐PCXR_PBSN‐E‐P_. Values in **bold** indicate the best performance.

Datasets	Micro F1 (%)	Macro F1 (%)	Weight F1 (%)	Sample F1 (%)	Subset ACC (%)	ACC_exam_ (%)	F1_exam_ (%)	One error (%)	Coverage
VinDr‐PCXR	66.1	16.5	53.8	60.9	60.9	60.9	60.9	39.3	1.8275
VinDr‐PCXR_ABSN_	66.3	**16.8**	**54.1**	62.1	62.0	62.0	62.1	38.8	1.7886
VinDr‐PCXR_PBSN‐E‐P_	**66.4**	15.3	53.4	**63.5**	**63.5**	**63.5**	**63.5**	**37.0**	**1.8304**

## 4 DISCUSSION

This paper proposes an efficient labeling approach for fine‐tuning a bone‐suppression network applicable to the CXR images of pediatric patients. The proposed approach involves three steps: distance transform–based bone‐edge detection, traditional image processing–based bone suppression, and fully automated pediatric bone suppression. The proposed approach has four main advantages. First, in contrast to data‐driven deep learning–based bone suppression networks, the proposed model‐driven traditional image processing–based bone suppression model is suitable for application to any CXR image. Second, compared with the thin plate spline surface‐fitting strategy^49^ used by Hogeweg et al.,^17^ the proposed Poisson reconstruction approach ensures that all the pixels in the nonbone regions equal 0, thereby ensuring the complete preservation of soft‐tissue information in these regions, which is unattainable through DES. Third, the proposed bone‐edge detection network reduces labor cost by eliminating the need for manually labeling pediatric CXR images; it takes 50 min to manually delineate bone edges in a CXR image but only 5 min to interactively extract bone‐edge lists for each CXR image (Table 6). Fourth, the fine‐tuned pediatric bone‐suppression network, together with the trained bone‐edge detection network, can achieve fully automated CXR bone suppression for pediatric patients.

The proposed method has two limitations. First, the proposed traditional image processing–based model assumes that bones are long and continuous, which is not suitable for labeling CXR images showing abnormal bone shapes, fractures, and other features. Therefore, the fully automated bone‐suppression network fine‐tuned on labeled pediatric CXR images cannot be used for patients with abnormal bone shape or fracture. Second, in contrast to previous CXR bone‐suppression approaches that produce DES‐style results, our approach produces bone images in which the pixels in the nonbone regions strictly have a value of 0, which may affect reader performance due to unfamiliar appearance.

Two tasks remain to be completed. First, as presented in Table 3, the proposed traditional image processing–based model was quantitatively evaluated only in terms of the RWC, which cannot provide evidence that the soft‐tissue structures behind the ribs, such as pulmonary vessels and airways, have been preserved. We plan to invite radiologists to quantitatively evaluate the preservation of soft‐tissue structures by asking them to subjectively compare and rate the bone‐suppression approaches mentioned in Table 6 on various criteria. A bone‐suppression approach's score can be calculated by adding weights to all items. Second, Table 6 indicates that PBSN‐E‐P achieved fully automated bone suppression for pediatric CXR images and had a unique advantage over PCA‐M and PCA‐D. However, the RWC values in Tables 3 and 5 indicate that PCA‐D and PBSN‐E‐P, which are based on deep learning, have relatively low generalizability. We plan to demonstrate that the benefits of automation achieved by PBSN‐E‐P are worth the sacrifice in generalizability. Moreover, we plan to use PCA‐D to label bone edges and images for additional CXR images of pediatric patients of different ages in P260_220unlabeled and thereby fine‐tune the bone‐edge detection network and bone‐suppression network. Ablation studies will also be conducted to investigate generalizability. Finally, we will use PCA‐M and PCA‐D to label the VinDr‐PCXR dataset, obtaining VinDr‐PCXR_PCA‐M_ and VinDr‐PCXR_PCA‐D_, respectively, and compare these datasets with VinDr‐PCXR, VinDr‐PCXR_ABSN_, and VinDr PCXR_PBSN‐E‐P_ (Table 7).

## 5 CONCLUSION

This paper proposes an efficient labeling approach for fine‐tuning a bone‐suppression network applicable to the CXR images of pediatric patients. The proposed approach involves three steps: distance transform–based bone‐edge detection, traditional image processing–based bone suppression, and fully automated pediatric bone suppression. The proposed distance transform–based bone‐edge detection network can effectively reduce the labor cost associated with the manual labeling of pediatric CXR images. The proposed traditional image processing–based bone‐suppression model can obtain pediatric bone images by combining multiple traditional image processing techniques. The proposed fully automated pediatric bone‐suppression network, together with the proposed distance transform–based bone‐edge detection network, can ultimately achieve the automatic acquisition of bone and soft‐tissue images solely from the CXR image of a pediatric patient. The evaluation results obtained on the VinDr‐PCXR dataset demonstrate that the proposed approach has the potential to promote the development of computer‐aided diagnosis with pediatric CXR images.

## CONFLICT OF INTEREST STATEMENT

The authors declare no conflicts of interest.

## Data Availability

The data that support the findings of this study are available from the corresponding author, Wenhui Wang, upon reasonable request.
